# Supplementary material for: Emotional and Physical Symptoms Following Intimate Partner Violence Victimization in the United States: Implications for Law and Public Health Policy
Source: Int J Environ Res Public Health. 2025 Dec 5;22(12):1829. doi: 10.3390/ijerph22121829 (PMC12732514; doi:10.3390/ijerph22121829)
Supplement: Supplementary file 1 [file ijerph-22-01829-s001.zip › ijerph-3903954-supplementary.pdf]

Table S1. Predicted Probabilities of Physical and Emotional Symptoms by Injury Present (Yes/No) and Race/Ethnicity

Outcome	Sex	Injury	Race	Predicted Probability (95% CI)
Physical symptom	Male	No Injury	American Indian/Alaska Native	0.476 (0.275–0.677)
			Asian/Pacific Islander	0.399 (0.203–0.596)
			Black	0.213 (0.138–0.289)
			Hispanic	0.211 (0.135–0.288)
			Multiracial	0.255 (0.153–0.357)
			White	0.255 (0.179–0.332)
		Injury	American Indian/Alaska Native	0.709 (0.515–0.903)
			Asian/Pacific Islander	0.640 (0.423–0.858)
			Black	0.423 (0.265–0.581)
			Hispanic	0.420 (0.262–0.578)
			Multiracial	0.480 (0.306–0.653)
			White	0.480 (0.326–0.634)
	Female	No Injury	American Indian/Alaska Native	0.757 (0.607–0.907)
			Asian/Pacific Islander	0.695 (0.523–0.867)
			Black	0.484 (0.381–0.586)
			Hispanic	0.481 (0.375–0.586)
			Multiracial	0.541 (0.412–0.670)
			White	0.541 (0.449–0.634)
		Injury	American Indian/Alaska Native	0.894 (0.803–0.986)
			Asian/Pacific Islander	0.860 (0.745–0.976)
			Black	0.715 (0.584–0.846)
			Hispanic	0.713 (0.581–0.844)
			Multiracial	0.760 (0.631–0.888)
			White	0.760 (0.647–0.873)
Emotional symptom	Male	No Injury	American Indian/Alaska Native	0.606 (0.367–0.844)
			Asian/Pacific Islander	0.537 (0.309–0.765)
			Black	0.432 (0.311–0.553)
			Hispanic	0.423 (0.303–0.543)
			Multiracial	0.460 (0.307–0.613)
			White	0.420 (0.312–0.527)
		Injury	American Indian/Alaska Native	0.748 (0.534–0.962)
			Asian/Pacific Islander	0.691 (0.465–0.917)
			Black	0.594 (0.420–0.769)
			Hispanic	0.585 (0.411–0.760)
			Multiracial	0.621 (0.434–0.808)
			White	0.582 (0.415–0.749)
	Female	No Injury	American Indian/Alaska Native	0.816 (0.664–0.969)
			Asian/Pacific Islander	0.769 (0.607–0.932)
			Black	0.686 (0.586–0.786)
			Hispanic	0.678 (0.576–0.78)
			Multiracial	0.710 (0.586–0.834)
			White	0.675 (0.583–0.767)
		Injury	American Indian/Alaska Native	0.896 (0.788–0.999)
			Asian/Pacific Islander	0.866 (0.742–0.990)
			Black	0.809 (0.698–0.920)

			Hispanic	0.803 (0.690–0.916)
			Multiracial	0.826 (0.710–0.941)
			White	0.801 (0.691–0.911)

Note. Survey-weighted logistic regression. Predictions averaged over years, age, region, and population size.

Table S2. Predicted Probabilities of Physical and Emotional Symptoms by Sex, Injury Present (Yes/No), and Race/Ethnicity

Outcome	Sex	Injury	Race	Predicted Probability (95% CI)
Physical symptom	Male	No Injury	White	0.255 (0.179–0.332)
			Hispanic	0.211 (0.135–0.288)
			Multiracial	0.255 (0.153–0.357)
			Asian/Pacific Islander	0.399 (0.203–0.596)
			American Indian/Alaska Native	0.476 (0.275–0.677)
			Black	0.213 (0.138–0.289)
		Injury	White	0.480 (0.326–0.634)
			Hispanic	0.420 (0.262–0.578)
			Multiracial	0.480 (0.306–0.653)
			Asian/Pacific Islander	0.640 (0.423–0.858)
			American Indian/Alaska Native	0.709 (0.515–0.903)
			Black	0.423 (0.265–0.581)
	Female	No Injury	White	0.541 (0.449–0.634)
			Hispanic	0.481 (0.375–0.586)
			Multiracial	0.541 (0.412–0.670)
			Asian/Pacific Islander	0.695 (0.523–0.867)
			American Indian/Alaska Native	0.757 (0.607–0.907)
			Black	0.484 (0.381–0.586)
		Injury	White	0.760 (0.647–0.873)
			Hispanic	0.713 (0.581–0.844)
			Multiracial	0.760 (0.631–0.888)
			Asian/Pacific Islander	0.860 (0.745–0.976)
			American Indian/Alaska Native	0.894 (0.803–0.986)
			Black	0.715 (0.584–0.846)
Emotional symptom	Male	No Injury	White	0.420 (0.312–0.527)
			Hispanic	0.423 (0.303–0.543)
			Multiracial	0.460 (0.307–0.613)
			Asian/Pacific Islander	0.537 (0.309–0.765)
			American Indian/Alaska Native	0.606 (0.367–0.844)
			Black	0.432 (0.311–0.553)
		Injury	White	0.582 (0.415–0.749)
			Hispanic	0.585 (0.411–0.760)
			Multiracial	0.621 (0.434–0.808)
			Asian/Pacific Islander	0.691 (0.465–0.917)
			American Indian/Alaska Native	0.748 (0.534–0.962)
			Black	0.594 (0.420–0.769)
	Female	No Injury	White	0.675 (0.583–0.767)
			Hispanic	0.678 (0.576–0.780)
			Multiracial	0.710 (0.586–0.834)
			Asian/Pacific Islander	0.769 (0.607–0.932)
			American Indian/Alaska Native	0.816 (0.664–0.969)

	Injury	Black	0.686 (0.586–0.786)
		White	0.801 (0.691–0.911)
		Hispanic	0.803 (0.690–0.916)
		Multiracial	0.826 (0.710–0.941)
		Asian/Pacific Islander	0.866 (0.742–0.990)
		American Indian/Alaska Native	0.896 (0.788–0.999)
		Black	0.809 (0.698–0.920)

Note. Survey-weighted logistic regression. Predictions averaged over year, age, region, and population size.

Table S3. Predicted Probabilities of Emotional and Physical Symptoms Among IPV Survivors with Injury by Sex, Probable TBI, and Race/Ethnicity

Outcome	Sex	Probable TBI	Race	Predicted Probability (95% CI)
Emotional symptom	Female	Yes	American Indian/Alaska Native	0.928 (0.851–1.00)
			Asian/Pacific Islander	0.906 (0.816–0.996)
			Black	0.863 (0.781–0.945)
			Hispanic	0.858 (0.774–0.942)
			Multiracial	0.876 (0.792–0.960)
			White	0.857 (0.776–0.937)
		No	American Indian/Alaska Native	0.896 (0.788–0.999)
			Asian/Pacific Islander	0.866 (0.742–0.990)
			Black	0.809 (0.698–0.920)
			Hispanic	0.803 (0.690–0.916)
			Multiracial	0.826 (0.710–0.941)
			White	0.801 (0.691–0.911)
	Male	Yes	American Indian/Alaska Native	0.815 (0.647–0.983)
			Asian/Pacific Islander	0.768 (0.582–0.955)
			Black	0.685 (0.534–0.836)
			Hispanic	0.677 (0.525–0.829)
			Multiracial	0.709 (0.550–0.868)
			White	0.674 (0.530–0.818)
		No	American Indian/Alaska Native	0.748 (0.534–0.962)
			Asian/Pacific Islander	0.691 (0.465–0.917)
			Black	0.594 (0.420–0.769)
			Hispanic	0.585 (0.411–0.760)
			Multiracial	0.621 (0.434–0.808)
			White	0.582 (0.415–0.749)
Physical symptom	Female	Yes	American Indian/Alaska Native	0.927 (0.862–0.992)
			Asian/Pacific Islander	0.902 (0.818–0.986)
			Black	0.788 (0.685–0.891)
			Hispanic	0.786 (0.683–0.889)
			Multiracial	0.824 (0.726–0.923)
			White	0.825 (0.739–0.910)
		No	American Indian/Alaska Native	0.894 (0.803–0.986)

		Asian/Pacific Islander	0.860 (0.745–0.976)
		Black	0.715 (0.584–0.846)
		Hispanic	0.713 (0.581–0.844)
		Multiracial	0.760 (0.631–0.888)
		White	0.760 (0.647–0.873)
Male	Yes	American Indian/Alaska Native	0.783 (0.625–0.941)
		Asian/Pacific Islander	0.725 (0.538–0.911)
		Black	0.520 (0.364–0.675)
		Hispanic	0.517 (0.362–0.672)
		Multiracial	0.577 (0.413–0.740)
		White	0.577 (0.433–0.721)
	No	American Indian/Alaska Native	0.709 (0.515–0.903)
		Asian/Pacific Islander	0.640 (0.423–0.858)
		Black	0.423 (0.265–0.581)
		Hispanic	0.420 (0.262–0.578)
		Multiracial	0.480 (0.306–0.653)
		White	0.480 (0.326–0.634)

Note. Survey-weighted logistic regression. Sample restricted to respondents reporting injury from IPV. 'Probable TBI' compares the loss of consciousness status. Predictions averaged over year, age, education, region, and population size.
